# Variable Isotopic Compositions of Host Plant Populations Preclude Assessment of Aphid Overwintering Sites

**DOI:** 10.3390/insects8040128

**Published:** 2017-12-05

**Authors:** Michael S. Crossley, Shawn A. Steffan, David J. Voegtlin, Krista L. Hamilton, David B. Hogg

**Affiliations:** 1 Department of Entomology, University of Wisconsin-Madison, 1630 Linden Dr., Madison, WI 53706, USA; steffan@entomology.wisc.edu (S.A.S.); dhogg@cals.wisc.edu (D.B.H.); 2 Illinois Natural History Survey, 1816 S. Oak St., Champaign, IL 61820, USA; dvoegtli@illinois.edu; 3 Wisconsin Department of Agriculture, Trade & Consumer Protection, 2811 Agriculture Dr., Madison, WI 53718, USA; krista.Hamilton@wisconsin.gov

**Keywords:** ^13^C, ^15^N, buckthorn, dispersal, overwintering, soybean aphid, stable isotopes

## Abstract

Soybean aphid (*Aphis glycines* Matsumura) is a pest of soybean in the northern Midwest whose migratory patterns have been difficult to quantify. Improved knowledge of soybean aphid overwintering sites could facilitate the development of control efforts with exponential impacts on aphid densities on a regional scale. In this preliminary study, we explored the utility of variation in stable isotopes of carbon and nitrogen to distinguish soybean aphid overwintering origins. We compared variation in bulk ^13^C and ^15^N content in buckthorn (*Rhamnus cathartica* L.) and soybean aphids in Wisconsin, among known overwintering locations in the northern Midwest. Specifically, we looked for associations between buckthorn and environmental variables that could aid in identifying overwintering habitats. We detected significant evidence of correlation between the bulk ^13^C and ^15^N signals of soybean aphids and buckthorn, despite high variability in stable isotope composition within and among buckthorn plants. Further, the ^15^N signal in buckthorn varied predictably with soil composition. However, lack of sufficient differentiation of geographic areas along axes of isotopic and environmental variation appears to preclude the use of carbon and nitrogen isotopic signals as effective predictors of likely aphid overwintering sites. These preliminary data suggest the need for future work that can further account for variability in ^13^C and ^15^N within/among buckthorn plants, and that explores the utility of other stable isotopes in assessing likely aphid overwintering sites.

## 1. Introduction

Since its introduction into the United States in 2000, the soybean aphid (*Aphis glycines* Matsumura) has posed a significant problem to soybean growers in the northern Midwest, spreading to 80% of U.S. soybean growing regions by 2004 [1,2,3,4], and causing yield-limiting damage to soybean by removing nutrients and transmitting viruses [5,6]. Average soybean aphid population densities have decreased since 2004, potentially due to a number of factors, including natural enemies and farm inputs, such as neonicotinoid seed treatments [7]. However, localized populations above economic threshold continue to occur in the northern Midwest [8,9,10].

**Figure 1:**
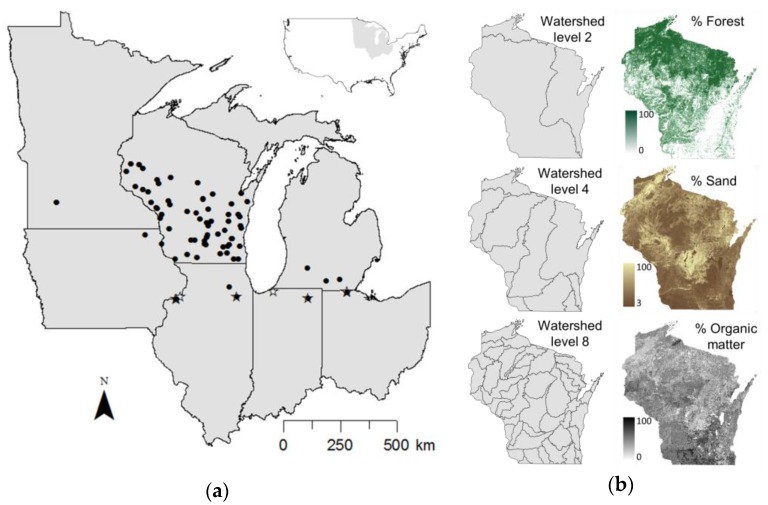
(**a**) Locations of *Rhamnus cathartica* leaf samples collected between May–June of 2015 (black circles; *n* = 90 sites) and known *Aphis glycines* overwintering sites (stars). Filled stars represent overwintering sites where buckthorn and aphid samples were obtained in 2015. (**b**) Environmental variables (% forest cover, % sand in surface soils, % organic matter in surface soils, watershed levels [corresponding to HUC2, HUC4, and HUC8]) used to detect associations with *R. cathartica* δ^13^C and δ^15^N among Wisconsin sites.

One challenge to soybean aphid management is the unpredictable nature of colonization patterns of soybean fields in the spring. It is known that soybean aphid in the United States utilizes common buckthorn (*Rhamnus cathartica* L.) as a primary, overwintering host [11]. However, despite enormous efforts to locate overwintering areas, few have been found (D.J.V., unpublished data), most occurring in isolated patches of buckthorn around 41 degrees latitude (Figure 1a). Furthermore, these confirmed overwintering locations have not consistently contained overwintered soybean aphids in the early spring. Still, soybean aphid continues to cover soybean fields of the northern Midwest in numbers that cannot be accounted for by known overwintering sites, raising several possibilities: (1) soybean aphid overwinters on another host plant, (2) overwintering locations vary geographically each year, and (3) potential overwintering areas are so vast that winter colonies continue to go unnoticed.

Genetic analyses suggest that long-distance dispersal is common in soybean aphid [12,13], and that soybean aphids at the few confirmed overwintering sites indeed colonize soybean in Wisconsin and other northern Midwest states [14]; but the detection of novel alleles among soybean aphids on their secondary host confirms that other overwintering source populations exist. Knowledge about overwintering locations of soybean aphid could aid management efforts: if a small number of overwintering locations consistently harbor a large proportion of founding aphid colonies, direct control efforts aimed at these locations would have exponential impacts on soybean aphid densities on a regional scale.

Tracking aphid dispersal to and from overwintering locations is challenging for several reasons: (1) the long-distance nature of aphid flight makes connecting locations of emigration to immigration all but guesswork [14], (2) there is not enough genetic structure for genetic markers to reliably distinguish sources and sinks [12,13], (3) aphids are too small and short-lived for monitoring with mark–recapture, radar, or radio-frequency identification, and (4) spring colonies on buckthorn are detectable for too short a time to be able to adequately scout a vast amount of area for colony presence.

Stable isotopes have proven useful for tracking animal migration under some circumstances [15,16,17]. For example, geographic variation in stable isotope composition of deuterium and ^13^C has been utilized to make inferences about monarch butterfly (*Danaus plexippus* L.) migration from overwintering sites in Mexico to summer habitats across a broad expanse of North America [18,19]. Within agricultural landscapes, variation in diet revealed by stable isotopes of ^13^C and ^15^N has been used to track movement of herbivores and predators among crop types [20,21,22,23,24]. The stable isotope composition of an organism derives from (1) the maternal contribution to its biomass; (2) the isotopic compositions of the proteins, lipids, water, and sugars in its diet; (3) N-turnover within the organism; (4) energy demands of the organism; and (5) background isotopic signatures, as a function of latitude/longitude, altitude, humidity, habitat type, and the myriad random effects of life in a heterogeneous landscape [25,26,27]. Thus, there are numerous, significant factors that can shape the isotopic composition of an organism. If the stable isotope composition of buckthorn is reliably transferred to feeding aphid colonies, and is geographically idiosyncratic or covaries with any of these background factors, such variability can be used to associate aphids arriving in soybean fields with their overwintering origins.

In this study, we quantified variation in stable isotopes of carbon (^13^C) and nitrogen (^15^N) in common buckthorn throughout Wisconsin, and from confirmed and potential overwintering locations of soybean aphid in Illinois, Iowa, Indiana, Michigan, Minnesota, and Ohio. We further investigated environmental variables that might explain variation in stable isotope content. We then compared ^13^C and ^15^N content of aphids collected from a subset of these locations with their host plants, to test if aphid sources could be inferred, based on similarity with host plant and region of origin.

## 2. Materials and Methods

Buckthorn was sampled at two spatial scales: a broad-scale sample encompassing areas with confirmed and potential overwintering sites in Illinois, Iowa, Indiana, Michigan, Minnesota, Ohio, and Wisconsin in May 2015. A finer-scale sample included buckthorn from 50 locations in 43 counties in Wisconsin between May–June 2015 (Figure 1a, Supplementary Table S1). At the broad-scale sample sites, three fully expanded leaves were taken from separate branches of one buckthorn plant. At the finer-scale sample sites, a hierarchical sampling scheme was implemented to quantify stable isotope variation within and among sample sites. To account for within-plant variation, three fully expanded leaves were taken from the terminal end of separate branches on one buckthorn plant, and another three fully expanded leaves were taken from a more basal position on those same branches. To account for within-site variation, leaves were sampled from two plants separated by at least 3 m. Leaves were frozen, dried, ground, and packaged in tin capsules for isotope analysis. Soybean aphids (alatae and apterae) were collected from a subset of buckthorn sample sites in May 2015 (Table 1). Aphids were stored in 100% ethanol at −20 °C, and entire, dried aphids were packaged in tin capsules for isotope analysis. Soybean aphid samples represent individuals from independent colonies except at Rock Island, where 12 aphids were sampled from the same colony.

Several stable isotopes have been used to investigate dispersal in terrestrial animals: stable isotopes of hydrogen and oxygen have been associated with gradients in annual precipitation, while those of carbon and nitrogen have been associated with soil biogeochemistry and plant physiology [15]. We focused analyses on carbon and nitrogen due to the fine-scale spatial resolution necessary for distinguishing geographic origins of soybean aphids based on buckthorn isotopic signatures. Quantification of ^13^C and ^15^N was done by the University of California-Davis Isotope Lab, using a PDZ Europa ANCA-GSL elemental analyzer interfaced to a PDZ Europa 20-20 isotope ratio mass spectrometer (Sercon Ltd., Cheshire, UK) with analytical error of 0.2 ‰ for ^13^C and 0.3 ‰ for ^15^N. Stable isotope content was represented in terms of δ^13^C (‰) and δ^15^N (‰) for statistical analyses. 

Several environmental variables were quantified at each sample site to test for an effect on stable isotope variation (Figure 1b): watershed, percent sand in the soil, percent soil organic matter in the soil, and percent forest. Watershed was used to account for any hydrological connectivity that could influence similarities in nutrient availability among sites. Percent sand was used to represent soil type, being correlated with other soil attributes, such as silt (R^2^ = −0.3) and clay (R^2^ = −0.5) content in the study extent. Soil organic matter was included due to its influence on nitrogen availability. Percent forest was used to represent landscape context; because forest and agriculture are the dominant land cover classes in the sampled area, percent forest gives some indication of the extent of agricultural inputs potentially affecting stable isotope variation. Watershed was determined from the United States Geological Survey (USGS) Watershed Boundary Dataset [28], and analysis was limited to Hydrologic Unit Codes (HUC) 2, 4, and 8. Percent sand and organic matter content within the sampling extent was derived from the Gridded Soil Survey Geographic Database [29] using the SSURGO OnDemand Dynamic Spatial Interpretations Tool [30]. Forest land cover data within the sampling extent was obtained from the USGS National Atlas land cover dataset [31] Percent sand, organic matter, and forest were calculated within a 1 km radius of each sample site using the “raster” and “rgdal” packages in R [32,33,34].

Statistical analyses were implemented in R using base functions, unless otherwise indicated. Correlations between aphid and buckthorn δ^13^C and δ^15^N values were assessed with linear regression; to maintain statistical independence among aphid samples, δ^13^C and δ^15^N values were averaged among the 12 Rock Island samples (Table 1). Differentiation among sites and states in δ^13^C and δ^15^N values was assessed with Tukey’s Honest Significant Differences (HSD). The effect of trivial variables (leaf position on plant and Julian date of collection), were evaluated using Analysis of Variance (ANOVA) and linear regression. Spatial autocorrelation among δ^13^C and δ^15^N values were evaluated using semivariograms implemented with the *variog* function of “geoR” [35]. Differentiation of stable isotope content among sites by environmental variables was assessed with ANOVA, linear regression, and non-metric multidimensional scaling (NMDS). Latitude, longitude, δ^13^C, and δ^15^N were standardized by subtracting the mean, and dividing by standard deviation prior to NMDS.

## 3. Results and Discussion

**Figure 2:**
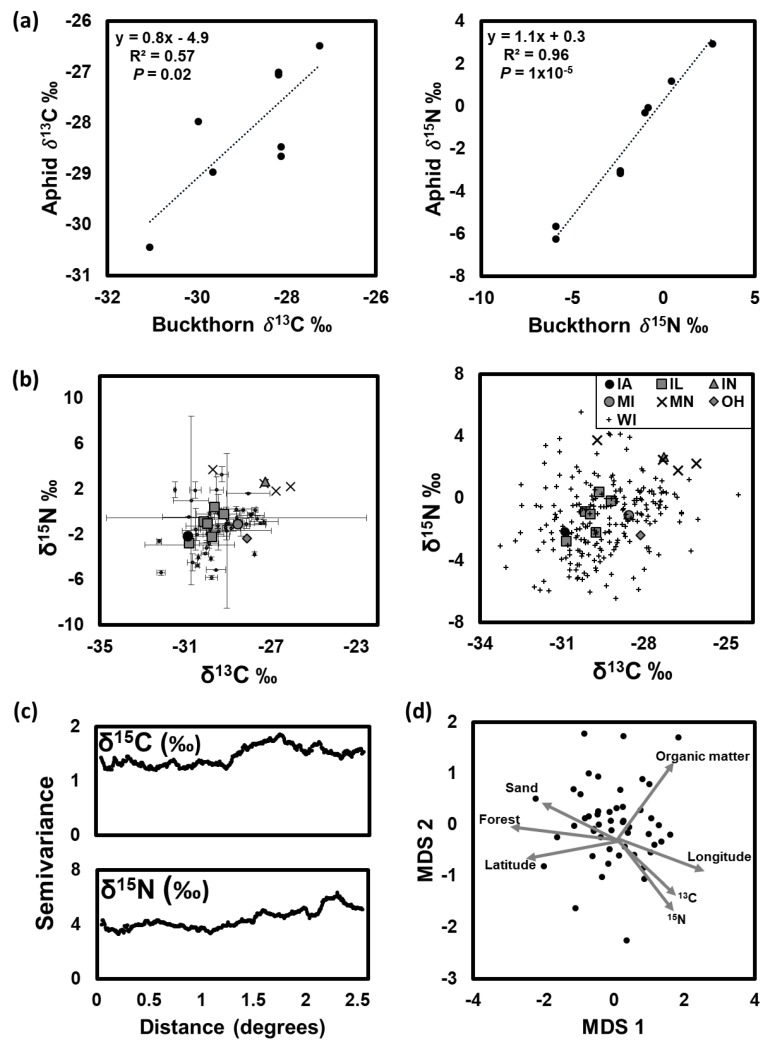
(**a**) Linear regressions of soybean aphid on buckthorn host plant δ^13^C and δ^15^N (‰; N = 11). A slope = 1 indicates perfect congruence between aphid and host plant, while a slope greater or less than 1 indicates enrichment or depletion, respectively, of the aphid relative to its host plant. (**b**) Comparison of individual (right) and mean ± standard deviation (left) δ^13^C and δ^15^N (‰) values of buckthorn plants. (**c**) Semivariograms depicting no spatial autocorrelation in δ^13^C and δ^15^N (‰) values among buckthorn plants in the Midwest at the scales sampled. (**d**) Non-metric multidimensional scaling plot depicting variability among buckthorn sample sites in terms of δ^13^C and δ^15^N (‰), and along environmental gradients in percent sand and organic matter content, percent forest, longitude and latitude. Stress was 0.21. The linear and non-metric fit (R^2^) between observed dissimilarity and ordination distance was 0.82 and 0.96, respectively.

Soybean aphid δ^13^C and δ^15^N values were significantly positively correlated with those of their host plants (Figure 2a), being slightly enriched (slope = 1.1), on average, relative to the plant in terms of ^15^N, and depleted (slope = 0.8) in terms of ^13^C. Previous studies have also found scaling of bulk ^15^N between aphids and host plants, though the direction of scaling varies among studies [24,36,37,38,39]. Importantly, the observed signal of isotopic congruence between the soybean aphids and buckthorn host plants suggests stable isotopes, particularly ^15^N, could be used to distinguish the origins of spring colonizers of soybean.

Variation in buckthorn δ^13^C and δ^15^N could not reliably distinguish buckthorn by site or even state of origin (Figure 2b), and there was no evidence for spatial autocorrelation in ^13^C or ^15^N within our study extent (Figure 2c). Tukey’s HSD in δ^13^C and δ^15^N between sites ranged from 0–5.8‰ and 0–9.1‰, respectively. Of the 2556 pairwise comparisons, 62 and 358 were significantly different (*p* < 0.05) for δ^13^C and δ^15^N, respectively. The majority of significantly different pairs involved comparisons of sites within Wisconsin (δ^13^C: 77%, δ^15^N: 88%). The remainder of significantly different pairs involved comparisons between Wisconsin and Minnesota or Illinois.

In the absence of site-level idiosyncrasy, associations between stable isotope composition and environmental variables could be used to infer regions of aphid origins. Environmental variables included in this study were: (1) latitude, (2) longitude, (3) watershed level (HUC 2, 4, and 8), (4) percent sand in the soil, (5) percent organic matter in the soil, and (6) percent forest. While variation in ^13^C was not correlated with any of the environmental variables (Table 2), ^15^N was related to percent sand and organic matter, which are themselves weakly negatively correlated in the landscape (*slope* = −1.48, R^2^ = 0.10, *F* = 6.28, *p* = 0.02). However, environmental differences among sites were not substantial enough, nor related to stable isotope variation enough to reliably distinguish geographic origins (Figure 2d). 

Accounting for sources of variation in stable isotope content could increase power to differentiate geographic locations by increasing the signal-to-noise ratio in ^13^C and ^15^N. Trivial variables included in this study included leaf position on plant (a surrogate for leaf age) and Julian date of collection. As expected, younger leaves were slightly enriched in ^13^C and ^15^N relative to older leaves (^13^C: difference in means = 0.45‰, *F* = 4.87, *p* = 0.03; ^15^N: difference in means = 0.58‰, *F* = 3.46, *p* = 0.06). Collection date had a weak but significant effect on ^13^C (*slope* = −0.03, *F* = 6.14, *p* = 0.02), and a negligible effect on ^15^N, suggesting differences in tissue age are more important than collection date (May–June) when accounting for trivial variation in comparisons of stable isotope variation among sites and regions. However, inclusion of trivial variables in regressions did not affect delineation of sites or states of origin based on ^13^C and ^15^N.

Our findings suggest that while the soybean aphid’s isotopic composition can be positively linked to that of its host plant, the isotopic signals of the buckthorn patches across the landscape were not distinct enough from one another to reliably trace the overwintering locations of soybean aphids that disperse to soybean fields. That said, our data are not definitive, and further sampling over more years would likely help to better address whether broad environmental factors (e.g., soil type, latitude/longitude, watershed) are affecting the isotopic composition of buckthorn enough to discern the aphids’ prior use of buckthorn. Because of the narrow host plant use by soybean aphids, and the patchy distribution of such host plants, it was anticipated that distinct isotopic signatures in the landscape would provide evidence of aphid overwintering sites. Stable isotopic signals can often provide evidence of less apparent, fine-scale differences in plant status or resource use. Fine-scale heterogeneity in ^13^C and ^15^N may arise from the natural isotopic mosaic of soils, mycorrhizal–plant associations, processes induced by soil microbiota, or exogenous nutrient inputs in the agricultural matrix. For example, if there were a large stand of leguminous plants at a given buckthorn site, nitrogen-fixing bacteria, which bring ^15^N signatures down to near-zero, could greatly impact the ^15^N signatures of the nitrate pools in the soil [40]. In addition, nitrogenous fertilizers applied to agroecosystems can have diverse manufacturing origins, overriding the effect of the local landscape on stable isotope content [41].

## 4. Conclusions

We found evidence that landscape heterogeneity, combined with fine-scale variation in isotopic signatures, appears to preclude the use of ^13^C and ^15^N as reliable indicators of soybean aphid geographic origin; but more data is clearly needed. Future work should (1) focus on better quantifying the variance of bulk ^13^C and ^15^N within buckthorn plants and among years, (2) draw from a larger sample of soybean aphids to quantify aphid–host plant scaling of stable isotope composition, and (3) explore the utility of other stable isotopes, such as ^87^Sr and ^208^Pb, which show variation among geological substrates [15], in assessing aphid overwintering sites.

## Figures and Tables

**Table 1:** Locations of *R. cathartica* plants from which *A. glycines* were collected during the spring of 2015.

State	County	Latitude	Longitude	Date	No. Aphids
Illinois	Rock Island	41.465	−90.577	12 May 2015	12 ^a^
	Will	41.520	−88.169	12 May 2015	1
Indiana	Noble	41.479	−85.349	13 May 2015	1
Ohio	Lucas	41.658	−83.782	13 May 2015	2 ^b^
Wisconsin	Dane	43.062	−89.445	15 May 2015	1
	Dodge	43.462	−88.639	23 May 2015	2 ^b^

^a^ δ^13^C and δ^15^N values of these aphid samples were averaged prior to statistical analysis; ^b^ These aphid samples were collected from different buckthorn plants.

**Table 2:** Summary statistics of linear regressions of stable isotope content (‰) on environmental variables potentially relevant to ^13^C and ^15^N variation in plants. Degrees of freedom = 48.

	Slope		R^2^		F-Statistic		*p* Value	
Variable	δ^13^C	δ^15^N	δ^13^C	δ^15^N	δ^13^C	δ^15^N	δ^13^C	δ^15^N
Latitude	−0.33	−0.57	0.03	0.03	2.36	2.32	0.13	0.13
Longitude	0.06	0.25	−0.01	0.01	0.30	1.60	0.58	0.21
Percent sand	0.53	−2.92	−0.01	0.07	0.42	4.61	0.52	0.04
Percent organic matter	0.06	0.66	−0.02	0.07	0.09	4.44	0.76	0.04
Percent forest	−0.53	−1.67	−0.01	0.01	0.51	1.73	0.48	0.19
Watershed (HUC2)	n/a ^a^	n/a	n/a	n/a	0.03	0.54	0.87	0.47
Watershed (HUC4)	n/a	n/a	n/a	n/a	0.04	0.52	0.85	0.48
Watershed (HUC8)	n/a	n/a	n/a	n/a	0.04	0.52	0.85	0.48

^a^ R^2^ not presented because watershed units represent a categorical variable.

## Supplementary Materials

The following are available online at www.mdpi.com/2075-4450/8/4/128/s1, Table S1: Buckthorn sample locations.
