# Supplementary material for: Variable Isotopic Compositions of Host Plant Populations Preclude Assessment of Aphid Overwintering Sites
Source: Insects. 2017 Dec 5;8(4):128. doi: 10.3390/insects8040128 (PMC5746811; doi:10.3390/insects8040128)
Supplement: Supplementary file 1 [file insects-08-00128-s001.pdf]

insects

MDPI

Communication

Variable Isotopic Compositions of Host Plant Populations Preclude Assessment of Aphid Overwintering Sites

Supplementary Table S1. Buckthorn sample locations.

State	County	Latitude	Longitude	Date
IA	Winneshiek	43.314	−91.796	5/12/2015
IL	Kane	41.801	−88.461	5/12/2015
IL	Rock Island	41.466	−90.548	5/12/2015
IL	Rock Island	41.465	−90.577	5/21/2015
IL	Will	41.520	−88.169	5/12/2015
IN	Noble	41.479	−85.349	5/13/2015
MI	Hillsdale	41.997	−84.629	5/14/2015
MI	Kalamazoo	42.371	−85.361	5/14/2015
MI	Lenawee	42.030	−84.099	5/14/2015
MN	Redwood	44.241	−95.316	6/1/2015
MN	Redwood	44.241	−95.302	6/1/2015
OH	Lucas	41.658	−83.782	5/13/2015
WI	Adams	43.783	−89.619	6/15/2015
WI	Adams	43.174	−89.990	5/25/2015
WI	Adams	43.940	−89.781	5/27/2015
WI	Barron	45.308	−92.034	6/17/2015
WI	Brown	44.502	−87.992	6/24/2015
WI	Buffalo	44.546	−91.684	6/16/2015
WI	Buffalo	44.253	−91.562	6/16/2015
WI	Calumet	43.905	−88.096	6/15/2015
WI	Calumet	44.209	−88.377	6/19/2015
WI	Chippewa	44.879	−91.308	5/27/2015
WI	Columbia	43.594	−89.306	5/29/2015
WI	Columbia	43.592	−89.307	5/29/2015
WI	Columbia	43.318	−89.328	5/18/2015
WI	Crawford	43.066	−91.136	5/11/2015
WI	Dane	43.265	−89.363	6/2/2015
WI	Dane	43.147	−89.435	5/20/2015
WI	Dane	43.052	−89.376	5/21/2015
WI	Dane	43.081	−89.427	5/20/2015
WI	Dane	43.062	−89.445	5/15/2015
WI	Dodge	43.352	−88.912	6/8/2015
WI	Dodge	43.462	−88.639	5/23/2015
WI	Dunn	45.205	−91.893	6/17/2015
WI	Eau Claire	44.776	−91.245	5/27/2015
WI	Fond du Lac	43.713	−88.482	5/26/2015
WI	Grant	42.636	−90.600	5/12/2015
WI	Green	42.681	−89.731	5/19/2015
WI	Green	42.673	−89.727	5/19/2015
WI	Green Lake	43.709	−89.168	5/29/2015
WI	Iowa	43.162	−89.839	6/2/2015

WI	Jackson	44.281	−90.868	6/8/2015
WI	Jackson	44.178	−90.823	6/8/2015
WI	Jefferson	42.979	−88.710	6/1/2015
WI	Juneau	43.985	−90.118	6/15/2015
WI	Kenosha	42.636	−88.091	6/2/2015
WI	La Crosse	43.792	−91.207	5/8/2015
WI	La Crosse	43.904	−91.146	5/19/2015
WI	Lafayette	42.756	−90.142	6/16/2015
WI	Manitowoc	44.276	−87.751	5/26/2015
WI	Marathon	44.795	−89.706	6/17/2015
WI	Marquette	43.674	−89.361	5/29/2015
WI	Milwaukee	42.991	−88.055	6/12/2015
WI	Monroe	44.944	−90.770	6/9/2015
WI	Ozaukee	43.520	−87.981	6/15/2015
WI	Pepin	44.606	−91.873	6/16/2015
WI	Pierce	44.699	−92.182	6/16/2015
WI	Polk	45.323	−92.361	6/17/2015
WI	Portage	44.345	−89.508	6/5/2015
WI	Racine	42.619	−88.279	5/4/2015
WI	Rock	42.789	−88.829	5/4/2015
WI	Saint Croix	45.115	−92.537	6/17/2015
WI	Sheboygan	43.849	−88.042	5/26/2015
WI	Sheboygan	43.586	−88.023	6/15/2015
WI	Treampealeau	44.101	−91.351	6/16/2015
WI	Treampealeau	44.070	−91.320	6/1/2015
WI	Vernon	43.488	−90.848	5/8/2015
WI	Walworth	42.796	−88.565	5/4/2015
WI	Washington	43.222	−88.382	5/13/2015
WI	Waukesha	42.989	−88.515	6/1/2015
WI	Waukesha	43.047	−88.466	6/12/2015
WI	Waupaca	44.480	−89.102	6/5/2015
WI	Waushara	44.047	−89.294	5/29/2015

© 2017 by the authors. Submitted for possible open access publication under the terms and conditions of the Creative Commons Attribution (CC BY) license (http://creativecommons.org/licenses/by/4.0/).
